# Assessing Oxygen Weaning Readiness in NICU Patients Using Oxygen Saturation Histograms: A Systematic Review

**DOI:** 10.7759/cureus.117368

**Published:** 2026-10-02

**Authors:** Phornphat Rasamimari, Ameer Khilfeh, Amy Pham, Luna Nasry, Shannon Murphy

**Affiliations:** 1 Pediatrics and Neonatology, University of Illinois Chicago, Chicago, USA; 2 Psychology, University of Michigan, Ann Arbor, USA; 3 Pediatrics, University of Illinois Chicago, Chicago, USA

**Keywords:** hyperoxia, nicu patients, oxygen saturation histograms, oxygen weaning, preterm infants, respiratory support, term infants

## Abstract

Weaning late preterm and term infants from respiratory support remains challenging because clinicians must balance the risks of hyperoxia and hypoxia while determining when room air trials and discharge are appropriate. Oxygen saturation histograms provide an objective method for evaluating oxygenation trends over time, but standardized approaches for neonatal intensive care unit (NICU) patients, particularly infants ≥34 weeks' gestation, remain limited. This systematic review evaluated whether oxygen saturation histogram patterns can predict readiness for room air trials or inform discharge decisions in NICU patients receiving nasal continuous positive airway pressure, high-flow nasal cannula, or low-flow nasal cannula oxygen therapy. Studies involving infants with major congenital anomalies, postmenstrual age greater than 42 weeks, or invasive mechanical ventilation, intubation, or tracheostomy extending beyond 36 weeks' postmenstrual age in the very low birth weight subgroup were excluded. A comprehensive literature search was conducted across multiple databases from July to December 2024, and two reviewers independently screened studies and assessed risk of bias using a modified Quality in Prognosis Studies (QUIPS) tool. Nine studies met the inclusion criteria. Across the available evidence, greater time within target oxygen saturation ranges was associated with readiness for oxygen weaning, whereas excessive time below or outside defined thresholds suggested the need for continued monitoring or respiratory support. However, study populations, saturation thresholds, monitoring durations, and outcome definitions varied substantially, preventing meta-analysis and limiting direct standardization. Oxygen saturation histograms appear promising as an adjunctive tool for guiding oxygen weaning and discharge decisions in NICU patients, but validated thresholds for late preterm and term infants are not yet established. Multicenter prospective studies using consistent histogram definitions, standardized room air trial criteria, and long-term outcome assessment are needed to support individualized and evidence-based respiratory care.

## Introduction and background

In neonatal intensive care units (NICUs), weaning infants from respiratory support remains highly variable and clinically uncertain. Existing evidence and weaning algorithms have focused largely on preterm infants, most often those born at less than 32 weeks' gestation, leaving little established, population-specific guidance for late preterm and term infants despite their distinct physiology and shorter expected respiratory course [1-3]. This gap is the focus of the present review: standardized, evidence-based criteria for oxygen weaning readiness in late preterm and term NICU infants are not yet established, and it remains unclear whether histogram-based approaches validated in more preterm populations are directly transferable to this group.

The clinical challenge lies in balancing the risks of both hyperoxia and hypoxia. Excessive oxygen exposure, often a result of prolonged invasive ventilation, is a known contributor to bronchopulmonary dysplasia (BPD) and retinopathy of prematurity (ROP). Conversely, intermittent or prolonged hypoxia has been associated with increased mortality, severe ROP, pulmonary hypertension, and neurodevelopmental delays [1,4,5]. Hypoxia-related events such as apnea, bradycardia, and desaturation significantly impact hospital length of stay, healthcare costs, and overall quality of life in both very low birth weight and late preterm or term infants [6,7].

Clinical targets vary by gestational age (GA) and disease status. For preterm infants on oxygen support, a saturation range of 90-95% is generally favored; for term infants, values above 94% are typically preferred. Yet, defining what constitutes "normal" on room air is less straightforward. In healthy term neonates, median oxygen saturation has been observed at 95.4%, with roughly one-quarter of infants spending a significant portion of time between 90-94% SpO2 [8]. These findings may provide a baseline for evaluating weaning readiness once pathologies are excluded.

Recent advances in histogram-based monitoring offer a promising tool for real-time, objective assessment of oxygenation trends. Unlike manual interpretation, histogram data enhances precision in maintaining saturation within target ranges, flagging episodes of extreme hypoxemia (<80%) or hyperoxia (>98%) [9]. Several studies have demonstrated that cumulative exposure to extreme hypoxemia, along with prolonged time spent in higher-risk histogram classes based on a histogram-derived classification system for preterm infants under 34 weeks' gestation, is associated with increased risks of severe ROP, mortality, and BPD [10,11].

Multiple studies have proposed clinical and histogram-based markers for oxygen weaning readiness in preterm infants. One study reported that successful weaning occurred in infants receiving fraction of inspired oxygen (FiO2) between 21-25%, nasal continuous positive airway pressure at 4-5 cmH2O, and who spent at least 60% of their time with oxygen saturation (SpO2) ≥95% [2]. Similarly, criteria such as <15% of time below 86% saturation and >50% above 96% were associated with positive outcomes [1]. While these findings are encouraging, they have yet to be extrapolated to infants at 34 weeks' GA or greater.

Current practice also benefits from emerging technologies, such as closed-loop oxygen controllers, which adjust FiO2 automatically based on continuous SpO2 feedback, minimizing caregiver workload while improving target maintenance [12,13]. Other physiologic indicators, such as monitor-recorded hypoxia exceeding 5% of the day or tachypnea over 30%, have been shown to predict clinical deterioration and poor weight gain [14]. Although feeding modality (bolus vs. continuous) does not generally impact oxygen instability at a population level, some infants display individual trends responsive to feeding pattern changes [15].

Despite ongoing advancements, a critical knowledge gap persists: although histogram-guided weaning has been used for decades, no standardized approach or best practice has been established for its implementation in NICU patients. Given the physiological maturity of this population and the variability in oxygen dependency and susceptibility to oxygen toxicity, particularly among very low birth weight infants, this gap remains a substantial barrier to optimizing care.

Therefore, the present study aims to investigate the relationship between oxygen saturation histogram parameters and readiness for oxygen weaning, as well as their potential role in informing discharge criteria for NICU patients. These findings will be instrumental in guiding safer, more personalized, and standardized care strategies for this vulnerable population.

Research question

Can trends in oxygen saturation histograms predict the success of room air trials or serve as part of the discharge criteria for NICU patients?

Aims

The aims of this study are twofold. Aim 1: To determine standardized trends in oxygen saturation histograms that can serve as indicators of readiness for room air trials in NICU patients. Aim 2: To determine the acceptable range of oxygen saturation histogram parameters that can be used as part of the discharge criteria for late preterm and term infants in the NICU.

## Review

Study design

This study comprises a systematic review conducted in accordance with the Cochrane Handbook for Systematic Reviews, encompassing steps such as planning the review, identifying and selecting relevant studies, extracting data, and assessing risk of bias [16]. Although a meta-analysis was originally planned as a second part of this study, substantial clinical and methodological heterogeneity across included studies precluded pooled quantitative synthesis. Institutional Review Board (IRB) approval was not required, as the study does not involve human or animal subjects. This review was not prospectively registered (e.g., with the International Prospective Register of Systematic Reviews (PROSPERO)), and no prior written protocol was published.

Eligibility criteria

Eligible studies were defined according to the following prespecified criteria.

Population

We included preterm, late-preterm, and term infants who were born or cared for in a NICU and were assessed at a postmenstrual age of 42 weeks or less. For studies that enrolled mixed populations or assessed infants at multiple time points, data were eligible only when results for infants meeting these criteria were reported separately or could be obtained from the study authors. We excluded infants with congenital anomalies likely to affect respiratory physiology or airway management, including airway malformations; infants with neuromuscular or neurological disorders likely to affect respiratory control; and, within the very low birth weight (<1500 g) subgroup, infants requiring invasive mechanical ventilation, intubation, or tracheostomy extending beyond 36 weeks' postmenstrual age (a marker of established, severe bronchopulmonary dysplasia). Studies were excluded when these participants could not be separated from the eligible population.

Respiratory Support and Exposure

Eligible infants were receiving noninvasive respiratory or oxygen support, either nasal continuous positive airway pressure (NCPAP), high-flow nasal cannula (HFNC), or low-flow nasal cannula (low NC), with continuous or near-continuous pulse oximetry (SpO2) monitoring summarized using a histogram-, distribution-, or trend-based method (e.g., time-in-range percentages, histogram classification systems, or nomograms) over a defined monitoring period. Studies that did not report a defined method of summarizing oxygen saturation data over time were excluded.

Outcomes

Eligible outcomes included readiness for, or success of, a room air trial; discontinuation of supplemental oxygen or noninvasive respiratory support; post-weaning respiratory stability (e.g., need for escalated support or weaning failure); and discharge readiness, as defined by the individual study authors.

Information sources

We conducted a systematic review and analysis of relevant English-language studies. In collaboration with a librarian specializing in systematic reviews, we searched Embase, MEDLINE via PubMed, the Cumulative Index to Nursing and Allied Health Literature (CINAHL), Web of Science, the Cochrane Library, and Google Scholar on the dates reported in the Supplementary Table, and we supplemented these searches with backward and forward citation searching. Database-specific strategies combined controlled vocabulary, where available, with free-text terms for three concepts: histograms; oxygen, oxygen saturation, oximetry, or weaning; and neonatal populations, including neonate, newborn, infant, preterm, prematurity, term, and full term. Searches were conducted between July and December 2024. English-language restrictions were applied as reported in the Supplementary Table; Google Scholar was additionally limited to publications through November 30, 2024. The Cochrane Library search retrieved 12 records; on manual review, 10 were duplicates of records already identified through the other databases, and two were confirmed ineligible, so the Cochrane Library contributed no unique records for screening. Citation searching was completed on December 30, 2024, and one citation was screened for eligibility.

Search strategy and selection process

All studies identified through the search algorithm were imported into a reference management program (EndNote, Clarivate, Philadelphia, PA) and a systematic review production platform (Covidence, Veritas Health Innovation, Melbourne, Australia). The screening process was conducted to identify studies meeting the predefined inclusion criteria. Initially, two independent reviewers conducted abstract screening, followed by full-text screening to assess study eligibility. Reasons for exclusion were recorded and reported using a PRISMA flow diagram [17].

Data collection process

All data were extracted from eligible studies and incorporated into Covidence and Excel (Microsoft Corporation, Redmond, WA). The outcome measures were recorded as discrete numbers to indicate count or sample size, with variables clearly defined. This process was conducted independently by two reviewers; any inconsistencies were resolved with the assistance of a biostatistician. Records and data were managed using Microsoft Word and Excel (Microsoft Corporation) and shared with the team. Extracted data included the following: title/authors, study design, date of study, number of subjects, maternal/neonatal factors, clinical conditions and/or interventions, number of subjects in control/study groups, demographic characteristics, and infant clinical course/intervention prior to outcome.

Risk of bias assessment

The modified Quality in Prognosis Studies (QUIPS) tool was used to assess risk of bias, with two reviewers conducting the evaluation [18]. The six domains assessed included bias due to study participation, attrition, prognostic factor measurement, outcome measurement, confounding, and statistical analysis and reporting. Each domain was rated as having high, moderate, or low risk of bias. Both reviewers independently assessed each study, and any discrepancies in domain-level ratings were resolved through consensus. Because the standard QUIPS tool assesses risk of bias at the domain level only, we extended it for this review by additionally assigning each study an overall risk-of-bias judgment, reached through consensus discussion between the two reviewers using the following prespecified criteria: studies were rated as low risk when all domains were rated low, or when domain-level concerns present - most commonly related to confounding, given the observational design of all included studies - were judged unlikely to meaningfully affect the prognostic factor-outcome relationship; as moderate risk when three or more domains had concerns judged capable of influencing the observed association; and as high risk when any domain contained serious methodological limitations judged likely to distort the prognostic factor-outcome relationship.

Synthesis methods

A quantitative meta-analysis, originally planned using a random-effects model in R (R Foundation for Statistical Computing, Vienna, Austria) with the metafor package, was not performed because the nine included studies did not provide a sufficiently comparable set of exposure-outcome relationships for valid pooling. The studies differed in gestational age, respiratory support modality, monitoring duration, histogram or exposure definitions, outcome definitions, and reported effect measures; consequently, no common effect measure could be derived without introducing potentially invalid assumptions. Instead, we conducted a narrative synthesis. Studies were grouped thematically according to their primary outcome domain: (1) predictors of weaning readiness or success, (2) post-weaning respiratory stability and discharge benchmarks, and (3) proposed thresholds and normative saturation ranges applicable to late preterm and term infants. Within each thematic group, studies were compared descriptively across population characteristics, respiratory support modality, monitoring duration, and the specific histogram or exposure metric used. Differences in outcome definitions were reported alongside each finding rather than harmonized into a common effect measure. Effect estimates, confidence intervals, and measures of predictive performance were reported when provided by the original studies; otherwise, findings were described using the thresholds and significance values reported by the study authors.

Results

The systematic review process is summarized using a PRISMA flow diagram (Figure 1). A total of 231 records were initially identified across six databases (Embase, MEDLINE/PubMed, Google Scholar, CINAHL, Web of Science, and the Cochrane Library) and citation searching, distributed as follows: Embase (n = 60), MEDLINE/PubMed (n = 58), CINAHL (n = 35), Web of Science (n = 30), Cochrane Library (n = 12), Google Scholar (n = 35), and citation searching (n = 1). Of the 12 Cochrane Library records, 10 were duplicates of records already identified through the other databases, and two were confirmed ineligible on inspection, so the Cochrane Library contributed no unique records to screening. After removing 110 records before screening (108 duplicates and two records excluded for other reasons), 121 studies were screened. Of these, 66 were excluded at abstract screening, leaving 55 studies for full-text assessment. During eligibility assessment, 46 additional studies were excluded, and ultimately, nine studies were included in the final review. Although review articles, case reports, case series, and meta-analyses were eligible for screening, none ultimately met full-text eligibility criteria, most commonly due to insufficient extractable histogram or outcome data, resulting in a final set of nine primary observational studies.

**Figure 1:**
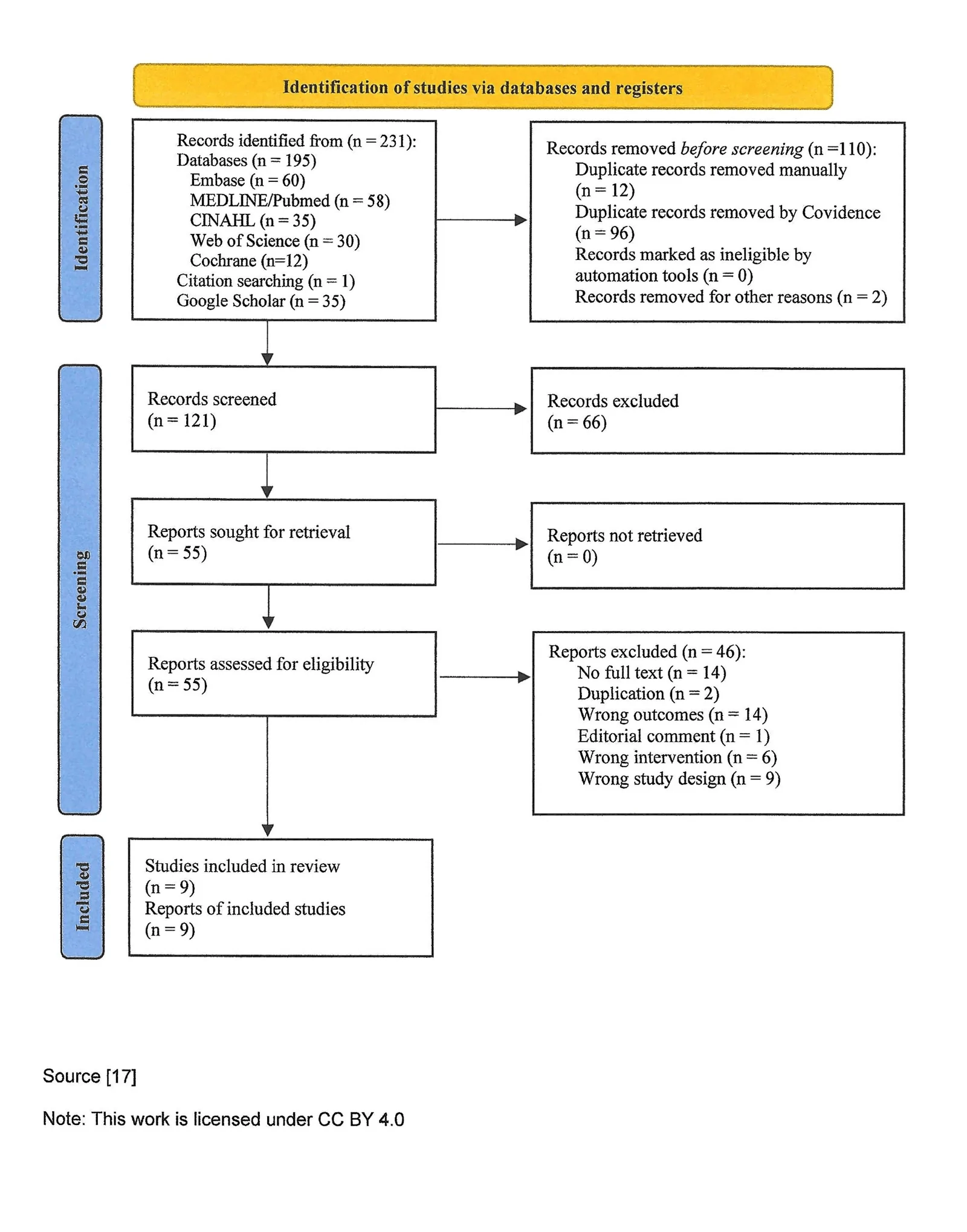
PRISMA flowchart of retrieved studies. PRISMA 2020 flow diagram of study identification, screening, and inclusion. A total of 231 records were identified through searches of five databases, including Embase (n = 60), MEDLINE/PubMed (n = 58), Cumulative Index to Nursing and Allied Health Literature (CINAHL) (n = 35), Web of Science (n = 30), and the Cochrane Library (n = 12), together with citation searching (n = 1) and Google Scholar (n = 35). Before screening, 110 records were removed (108 duplicates and two records excluded for other reasons, including 10 Cochrane Library records identified as duplicates of records already retrieved through other databases and two confirmed ineligible on manual review), leaving 121 records for title/abstract screening. Of these, 66 were excluded, and 55 reports were sought for retrieval (all 55 were retrieved) and assessed for eligibility. Forty-six reports were excluded at full-text review (no full text available, n = 14; duplication, n = 2; wrong outcomes, n = 14; editorial comment, n = 1; wrong intervention, n = 6; wrong study design, n = 9), leaving nine studies (nine reports) included in the review. Adapted from the PRISMA 2020 flow diagram template [17].

Tables 1, 2 summarize the QUIPS risk-of-bias assessment and descriptive characteristics of the nine included studies. Across the six QUIPS domains, only study 3, Gentle et al. (2020) [4], was judged to have a potentially moderate overall risk of bias; the remaining eight studies were rated low risk. Study designs, populations, gestational ages, and sample sizes varied considerably, ranging from single-center prospective cohorts of 30-101 infants to one larger retrospective cohort of 727 infants (Table 2).

**Table 1:** Risk-of-bias assessment using the modified Quality in Prognosis Studies (QUIPS) tool across nine included studies. D1: Study participation. D2: Attrition. D3: Prognostic factor measurement. D4: Outcome measurement. D5: Confounding. D6: Statistical analysis and reporting. Overall risk-of-bias judgment is provided for each study. Study #3 (Gentle et al., 2020) [4] was identified as having a potentially moderate risk of bias.

Study	Author (Citation)	D1	D2	D3	D4	D5	D6	Overall
1	Borenstein-Levin et al., 2020 [19]	Low	Low	Low	Low	Low	Low	Low
2	Borenstein-Levin et al., 2023 [11]	Low	Low	Low	Low	Moderate	Low	Low
3	Gentle et al., 2020 [4]	Moderate	Moderate	Moderate	Moderate	Moderate	Moderate	Moderate
4	Goetz et al., 2024 [8]	Low	Moderate	Low	Low	Moderate	Low	Low
5	Harigopal et al., 2011 [20]	Low	Low	Low	Low	Moderate	Low	Low
6	Mascoll-Robertson et al., 2016 [1]	Low	Low	Low	Low	Moderate	Low	Low
7	Mohsen et al., 2022 [21]	Low	Low	Low	Low	Moderate	Low	Low
8	Rath and Kluckow, 2016 [22]	Low	Low	Low	Low	Moderate	Low	Low
9	Warburton et al., 2019 [14]	Low	Low	Low	Low	Moderate	Moderate	Low

**Table 2:** Descriptive comparable table of nine studies based on the QUIPS assessment. AUROC, area under the receiver operating characteristic curve; BW, birth weight; FiO2, fraction of inspired oxygen; GA, gestational age; NICU, neonatal intensive care unit; QUIPS, Quality in Prognosis Studies; SpO2, peripheral oxygen saturation.

Study	Author	Published year	Country	Type of study	Study periods	Sample size	Gestation or birth weight	Domain 1 (Study participation)	Domain 2 (Attrition)	Domain 3 (Prognostic factor)	Domain 4 (Outcome measurement)	Domain 5 (Confounding)	Domain 6 (Statistical analysis)
1	Borenstein-Levin [19]	2020	Israel	Prospective cohort	6/2015 to 11/2015	73	≤34 weeks	Clear inclusion and exclusion criteria with robust representation.	Of 89 eligible infants, 16 were excluded with no substantial loss to follow-up.	O_2_ saturation instability was quantified via a novel five-type histogram. Continuous data sampling and standardized classification boosted accuracy and reproducibility.	Outcomes were objectively defined and stratified by histogram type. Analysis covered both cross-sectional and longitudinal perspectives.	Demographic comparisons were reported, but statistical adjustment for confounding variables was limited. No multivariate modeling was applied.	Utilized descriptive statistics, t-tests, chi-square tests, and visual data plots appropriately. Findings were statistically significant where applicable, and confidence intervals were used in range comparisons.
2	Borenstein-Levin [11]	2023	Netherlands	Retrospective cohort	12/2013 to 6/2017	61	<1500 g	Clear clinical criteria in a single level IV NICU.	All infants had valid O_2_ saturation data across four histogram intervals. Transparent reporting of patient flow supports completeness and reliability.	Histogram types were derived from standardized classification. Oxygenation instability was objectively quantified pre- and post-intervention.	Response to therapy is defined as a decrease in histogram type. Outcomes like need for invasive mechanical ventilation within 72 hours were clearly stratified across histogram categories, enhancing interpretability.	No multivariate modeling was performed to adjust for potential confounding influences.	Appropriate use of Wilcoxon rank-sum, Fisher’s exact, and chi-square tests. Predictive value metrics were calculated. P-values and boxplots enriched statistical transparency.
3	Gentle [4]	2020	USA	Prospective case-control	10/2016 to 8/2018	36	<30 weeks or <1250 g	Clear eligibility criteria from a regional NICU; however, limited generalizability due to single-center design and slightly small sample size.	Excluded appropriate cases but missed analysis of follow-up loss and bias.	Clearly defined prognostic factor tracked by histograms, but interpretations were subjective and may be skewed by short averaging.	Well-defined outcome tracked over time but lacked blinding and specifics on cause of death.	Baseline data provided, but no statistical adjustment or confounding variables; absence of multivariate analysis weakens causal inferences.	Used appropriate control charts and basic tests; however, absence of regression modeling or effect size reporting limits depth of analysis.
4	Goetz [8]	2024	USA	Prospective cohort	2/2020 to 6/2021	75	37 weeks 0 days to 41 weeks 6 days	Clearly defined inclusion and exclusion criteria; strong internal validity due to narrow eligibility focused on complicated deliveries and well infants.	All enrolled completed monitoring; no reported dropouts during the study period.	Oxygen saturation was measured with validated high-resolution pulse oximetry, and exclusion of low-signal quality data enhanced accuracy.	The primary outcome was the distribution of oxygen saturation intervals across five predefined ranges; the secondary outcome included short-term follow-up at 2 months. Clear definitions and rigorous data filtering improved reliability.	Baseline demographics and delivery variables were analyzed for association with oxygen saturation distribution, but limited adjustment for sleep and feed status or non-recorded physiological influences. No multivariable modeling applied.	Descriptive statistics and confidence intervals; appropriate visualizations; exploratory subgrouping into “high” vs. “low” oxygen saturation groups added interpretive depth.
5	Harigopal [20]	2011	UK	Prospective cohort	NA	43	29 weeks to 36 weeks	Well-defined inclusion and clear exclusion criteria.	All enrolled infants were included in final analyses with complete oxygen saturation and heart rate recordings.	Oxygen saturation profiles measured with standardized equipment over a 4-hour quiet period; rigorous artifact exclusion; use of validated software (PODS) for cumulative analysis.	Outcome was a reference range, not a health event, and clearly constructed using statistical percentiles and graphs; definitions consistent with clinical expectations.	Some baseline characteristics reported, but limited analysis on how they might influence SpO_2_; no stratification or adjustment for postnatal age or feeding status.	Descriptive statistics well-presented; cumulative distribution graphs offer clinical interpretability; software validated as a CE-marked device; appropriate methodology for reference range construction.
6	Mascoll-Robertson [1]	2016	USA	Prospective cohort	4/2012 to 7/2013	31	24 weeks to 32 weeks	Clear inclusion and exclusion criteria and protocol-aligned respiratory support ensured internal consistency.	Only 4 were excluded due to missing data, transfer, or no transition attempt. The final analysis included all eligible and transitioned infants. The flow diagram enhanced transparency.	The primary factor was objectively measured with validated pulse oximetry. Histogram-based data enhanced precision and reproducibility.	Outcome: success was assessed independently of histogram data. Outcome definition matched clinical best practice.	Some baseline comparisons were provided between success and failure groups, but no regression modeling or adjustment for confounding variables. Interpretive confidence is limited as a result.	Applied appropriate statistics and tests with clear reporting; graphs enhanced clinical clarity.
7	Mohsen [21]	2022	Canada	Retrospective cohort	1/2015 to 12/2019	727	<29 weeks	Clear inclusion and exclusion criteria. Large sample size and well-defined setting with consistent weaning protocols enhancing internal validity.	254 infants excluded for reported reasons, but attrition and retrospective design may limit generalizability.	Oxygen saturation histograms tracked consistently every 6 hours using standardized monitoring practices. Histogram variables were clearly defined and statistically analyzed.	The primary outcome is clinically meaningful and consistently applied across groups. However, no blinding of outcome assessors.	Multivariable regression was used, adjusting for GA, sex, and saturation metrics. However, additional confounders like severity of lung disease, infection status, and feeding practices were not included in modeling.	Multivariate logistic regression and AUROC were used appropriately. Clear reporting of odds ratios, CIs, and p-values. Appropriate stratification by GA and BW; subgroup trends visualized with graphs and tables.
8	Rath [22]	2016	Australia	Prospective cohort	7/2014 to 2/2015	30	Full-term or term equivalent preterm.	Inclusion and exclusion criteria were clearly stated and aligned with goals of comparing saturation profiles across groups.	No dropout reported; data completeness was supported by high-quality signal filtering and robust device validation.	Continuous SpO_2_ monitoring was performed every 2 seconds using a validated device. Clear threshold stratification and signal filtering for motion/ambient light artifacts improved precision.	Outcome was clearly stated. Feeding periods were defined clearly. Though not prognostic per se, comparisons were systematic and clinically relevant.	Basic demographic data were balanced across groups. However, confounding variables like sleep cycles, feeding method, and prior minor respiratory instability were not statistically adjusted. Sample size limits multivariate analysis.	Used Mann-Whitney and Wilcoxon tests appropriately given skewed distributions. P-values were clearly reported. Saturation distribution graphs helped visualize comparative trends, though broader modeling was absent.
9	Warburton [14]	2019	USA	Prospective cohort	11/2016 to 3/2017	101	<36 weeks	Clear criteria for initiating room air or low-flow nasal cannula trials. Exposure and outcome parameters were well specified, focusing on FiO_2_ reduction patterns.	Only 7 infants were excluded; the rest were followed throughout the weaning period. Attrition-related bias appears minimal based on transparent reporting.	Objective markers were continuously monitored. Measurements were consistent and validated through clearly defined failure criteria, improving exposure classification.	The primary outcome was defined based on duration off support and physiologic stability. Subgroup analysis based on gestation strengthened clinical relevance.	Key prognostic factors were identified but not adjusted for in statistical modeling. Absence of stratified or multivariate analysis limits control over confounding influences.	Statistics were shown, but without modeling or confidence intervals, limiting precision despite biological plausibility.

Table 3 summarizes the histogram method, threshold, and outcome reported by each included study. We synthesize these findings below according to this review's two prespecified aims rather than restating them study by study.

**Table 3:** Comparative summary of included studies on oxygen saturation histogram use in NICU oxygen weaning, with reported statistical and predictive-performance measures. ANOVA, analysis of variance; AUC, area under the curve; AUROC, area under the receiver operating characteristic curve; BW, birth weight; CI, confidence interval; CPAP, continuous positive airway pressure; FiO₂, fraction of inspired oxygen; HFNC, high-flow nasal cannula; IMV, invasive mechanical ventilation; N, sample size; NCPAP, nasal continuous positive airway pressure; nCPAP, nasal continuous positive airway pressure; NPV, negative predictive value; OR, odds ratio; O₂, oxygen; PPV, positive predictive value; ROC, receiver operating characteristic; ROP, retinopathy of prematurity; RR, respiratory rate; SpO₂, peripheral oxygen saturation.

Author (year)	N	Population	Key histogram threshold	Outcome	Statistical/predictive-performance measures reported
Borenstein-Levin et al. (2020) [19]	73	Preterm infants, birth to discharge	5 histogram classification types	Reference framework for saturation instability quantification across NICU course	Student's t-test, χ²/Fisher's exact test. One 95% CI reported (mean difference in time >95%, type 1 vs. types 2–5: 24%, 95% CI: 20–28). Other group comparisons (e.g., days off respiratory/O2 support) reported as p-values only (p = 0.003-0.004). No OR, RR, or AUC reported.
Borenstein-Levin et al. (2023) [11]	61	Preterm infants post-Doxapram	5 histogram classification types	Histogram classification useful for evaluating treatment response; confirms saturation patterns post-intervention	Wilcoxon rank-sum/signed-rank test, Fisher's exact, χ². Positive and negative predictive values reported: PPV = 0.46, NPV = 0.94 for 24-h response predicting no IMV need within 72 h (NPV range = 0.88-0.94, PPV range = 0.40-0.46 across 4-, 12-, and 24-h response windows). No OR or AUC reported.
Gentle et al. (2020) [4]	36	Preterm infants on NCPAP	≥60% time SpO₂ ≥95%; FiO₂ = 21-25%; NCPAP = 4-5 cmH₂O	Histogram-based criteria associated with NCPAP wean success; high risk of bias noted	Quality-improvement, pre/post design analyzed with statistical process control charts (special-cause variation), χ², one-way ANOVA, Kruskal-Wallis with Dunn's test. Outcome rates compared descriptively (e.g., death/severe ROP 32.1%→18.0%; goal saturations 48.7%→57.6%, p < 0.0001). No OR, CI, or AUC reported — design does not produce these.
Goetz et al. (2024) [8]	75	37 weeks 0 days – 41 weeks 6 days	Median SpO₂ = 95.4%; time distribution: 0.07% ≤80%, 0.15% = 81-84%, 0.88% = 85-89%, 26.9% = 90-94%, 67.3% = 95-100%	Provides normative saturation profiles for term infants; useful comparison for NICU histogram interpretation	Linear model with robust standard errors; results expressed as ratio of median odds of SpO₂ in the 95-100% range. Female sex: ratio 2.46 (95% CI: 1.15-5.28, p = 0.02). Gestational age, birth weight, race, delivery mode, and bilirubin were not significant (ratios and 95% CIs reported for each in the original Table 2).
Harigopal et al. (2011) [20]	43	Healthy preterm infants	4-hour SpO₂ nomogram	Innovative real-time assessment tool; limited generalizability due to sample size	Descriptive construction of a reference range (median, 5th and 95th percentiles; cumulative frequency curve). No inferential statistics, effect estimates, confidence intervals, or measures of predictive performance were reported.
Mascoll-Robertson et al. (2016) [1]	31	Preterm NICU infants	<15% time below 86% SpO₂ in 24 hours	Associated with weaning failure; combined clinical + histogram criteria improved weaning success	χ², t-test. ROC-derived sensitivity/specificity reported at two thresholds: SpO₂<86% for ≥15% of time — 29% sensitivity, 96% specificity; ≥10% of time — 43% sensitivity, 83% specificity (no AUC value given). Spearman's ρ=0.585 (p=0.01) and ρ=−0.431 (p=0.035) for correlation with FiO₂ change. No OR or CI reported.
Mohsen et al. (2022) [21]	727	Preterm <30 weeks	>90% time with SpO₂ >89% (AUC 0.68)	Successful CPAP/HFNC wean maintained off support ≥1 week	Multivariable logistic regression. Adjusted OR for gestational age = 1.13 (95% CI: 1.03-1.24, p = 0.01); adjusted OR for % time SpO₂ > 89% = 1.08 (95% CI: 1.05-1.11, p < 0.001). AUROC = 0.68 (95% CI: 0.64-0.72) for % time SpO₂>89% predicting successful first trial off nCPAP.
Rath and Kluckow (2016) [22]	30	Term vs. preterm (term-equivalent)	Term: 0% time <90%, 4% time 90-95%; Preterm: 4% time <90%, 8% time 90-95%	Supports tailored saturation targets by gestational age; term benchmarks ≠ preterm	Mann-Whitney U-test, Wilcoxon signed-rank test. Group differences reported as p-values only (e.g., time <90%: p = 0.005; time 85-89%: p = 0.005; time 81-84%: p = 0.023). No OR, CI, or AUC reported.
Warburton et al. (2019) [14]	101	Premature infants	SpO₂ <90% for >5% of time; RR >70 bpm for >20% of time	Associated with need for escalated respiratory support within 3 days; >30% elevated RR → poor weight gain	ROC analysis: AUC = 0.814 (95% CI: 0.79-0.83) for SpO₂ < 90% predicting escalation within 3 days; AUC = 0.652 (95% CI: 0.63-0.68) for RR > 70. Multivariable logistic regression (adjusted OR): hypoxia 5-10% of time = 3.70 (95% CI: 2.05-6.70); hypoxia >10% = 4.56 (95% CI: 2.80-7.43); tachypnea >30% = 2.81 (95% CI: 1.69-4.67); all p < 0.0001.

Aim 1: Histogram Trends Indicating Readiness for Room Air Trials

Six of the nine studies addressed this aim [1,2,11,19-21]. A recurring pattern emerged across all six: infants who spent a greater proportion of time within a saturation range of approximately 90-95% were consistently more likely to wean successfully, while greater time below 86-90% was associated with weaning failure or post-weaning instability. This pattern held despite differences in histogram methodology - a five-type classification system [11,19], time-in-range percentages [1,2,21], or a real-time nomogram [20] - suggesting the direction of the association, though not a specific numeric cutoff, is reasonably consistent across measurement approaches. One study reported a formal measure of predictive performance (area under the curve = 0.68) [21], indicating modest discrimination.

Aim 2: Histogram Parameters for Discharge Criteria in Late Preterm and Term Infants

Only two included studies directly addressed infants ≥34 weeks' gestational age. Goetz et al. reported a median saturation of 95.4% in healthy term infants, with the largest proportion of monitored time (67.3%) spent at 95-100% saturation and a smaller proportion (26.9%) spent at 90-94% [8]. Rath and Kluckow found that term infants spent virtually no time below 90% saturation, compared with modest but measurable time below 90% in preterm-equivalent infants [22]. These two studies provide preliminary normative benchmarks for this population but represent a small evidence base relative to the six studies addressing Aim 1, most of which enrolled predominantly preterm infants.

Discussion

This review examined whether trends in oxygen saturation histograms can indicate readiness for room air trials and inform discharge criteria in NICU patients, with particular attention to late preterm and term infants (≥34 weeks' gestational age). The results above point to a consistent directional signal - more time in a 90-95% saturation range - but also reveal how limited the evidence becomes once narrowed to the population this review was designed to address.

Evidence for Weaning Readiness

The convergence across studies using different histogram methodologies strengthens confidence that the underlying association is not an artifact of any single measurement approach. However, consistency in direction does not equate to consistency in actionable thresholds: the specific cutoffs used to define "sufficient time in range" varied by several percentage points across studies, and none has been externally validated in a population distinct from the one in which it was derived. Todd et al. (not among the nine included studies, cited here as corroborating context) similarly emphasized the need for monitoring beyond the initial weaning phase [3], reinforcing that a single threshold assessed at one time point is unlikely to be sufficient on its own.

Evidence in Late Preterm and Term Infants

As the Results indicate, only two of the nine included studies directly studied infants ≥34 weeks' gestational age - a meaningful limitation given this review's stated aims. Literature not meeting formal inclusion criteria offers useful normative context: Shah et al. found that healthy late preterm and term infants spent approximately 7% of their time at saturations ≤90% [23], and Hunt et al.'s Collaborative Home Infant Monitoring Evaluation (CHIME) cohort data suggest that brief desaturations and periodic breathing may reflect normal physiology rather than pathology in this population [24]. This context helps distinguish expected variation in healthy late preterm and term infants from the more pathologic patterns described in very preterm infants, though it was not part of this review's systematic evidence base. The disparity between the six studies addressing weaning readiness broadly (predominantly in preterm infants) and the two studies specific to late preterm and term infants is, in our view, the central gap this review identifies. This gap is compounded by differences in respiratory support and study aims among the nine included studies: Borenstein-Levin et al. (2020) [19] and Warburton et al. (2019) [14] included infants who received invasive mechanical ventilation for part of their clinical course, in contrast to this review's focus on noninvasive support. Both cohorts' ventilated infants were assessed well before this exclusion threshold: all Borenstein-Levin et al.'s (2020) [19] subjects were assessed at less than 34 weeks' postmenstrual age, and no Borenstein-Levin et al.'s (2023) [11] subject was assessed beyond 36 weeks' postmenstrual age, so their inclusion is consistent with the eligibility criteria. Borenstein-Levin et al. (2020, 2023) primarily addressed development of the histogram classification system and its use in tracking doxapram response, respectively, rather than weaning readiness itself [11,19]. Two further studies (Goetz et al., 2024; Harigopal et al., 2011) established normative SpO2 histogram profiles in infants already breathing room air rather than during an active weaning trial [8,20]. These studies were retained because they provide relevant histogram methodology and physiological data in the absence of a larger body of directly applicable evidence, but their inclusion means that much of this review's evidence base is indirect and extrapolated rather than specific to the weaning process in the target population.

Clinical Applicability and Proposed Thresholds

Literature outside the nine included studies helps contextualize the clinical stakes of these thresholds. Sustained hypoxemia has been linked to pulmonary hypertension [25-27] and to lower neurodevelopmental scores at 12-24 months [28], while preterm infants' immature antioxidant systems increase vulnerability to hypoxic injury [7]. A 20% threshold for time spent outside a target saturation range has been proposed elsewhere as a pragmatic, evidence-informed benchmark rather than an established guideline [29]. Drawing on this context together with the pattern identified across our included studies, we present a set of candidate parameters (Table 4) and, separately, a proposed threshold pathway illustrating one possible gestational-age-stratified approach to applying these concepts in practice (Figure 2); Figure 2 is an independent, hypothesis-generating pathway rather than a visual restatement of Table 4. These are hypothesis-generating, not validated: they are derived from a small number of heterogeneous, observational studies with differing histogram definitions and outcome measures, and should not be interpreted as clinical practice recommendations.

**Table 4:** Histogram-based oxygen saturation thresholds: candidate parameters in infants without apnea or bradycardia at sea level*. * As altitude level affects oxygen tension, please be mindful to modify thresholds accordingly [30,31]. These guidelines are proposed based on available evidence and are currently unvalidated. They should be applied with clinical judgment as an adjunctive tool alongside established clinical criteria. SpO2, peripheral oxygen saturation; FiO2, fraction of inspired oxygen; NCPAP, nasal continuous positive airway pressure.

Parameter	Threshold	Clinical interpretation	Recommended action	Supporting citation
Time above SpO_2_ 89%	≥90% of monitoring period	Indicator of weaning readiness	Consider initiating room air trial	[21]
Time below SpO_2_ 86%	≤15% of 24-hour period	Acceptable lower-limit exposure	Monitor closely; reassess if threshold exceeded	[1,3]
Time outside target SpO_2_ range	≤20% of monitoring period	Clinically tolerable deviation	If >20%, adjust FiO_2_ or respiratory support promptly	[29]
Time SpO_2_ <90%	≤5% of monitoring period	Elevated risk of respiratory escalation and pulmonary hypertension	Escalate support; reassess weaning readiness	[14,25,26]
Time SpO_2_ <80%	Minimize; avoid sustained episodes >1 minute	Profound hypoxemia associated with increased risk of adverse outcomes	Immediate assessment and intervention, with reassessment of the oxygen strategy	[7,19]
Time SpO_2_ ≥95%	≥60% of monitoring period (on NCPAP)	Associated with NCPAP wean success	May proceed with weaning trial if clinical criteria met	[2]
Acute SpO_2_ drop ≥10% below stable baseline, sustained <90% >5 sec	Part of normal physiology if isolated	May represent periodic breathing; monitor trend	No intervention if isolated; track frequency and duration	[24]

**Figure 2:**
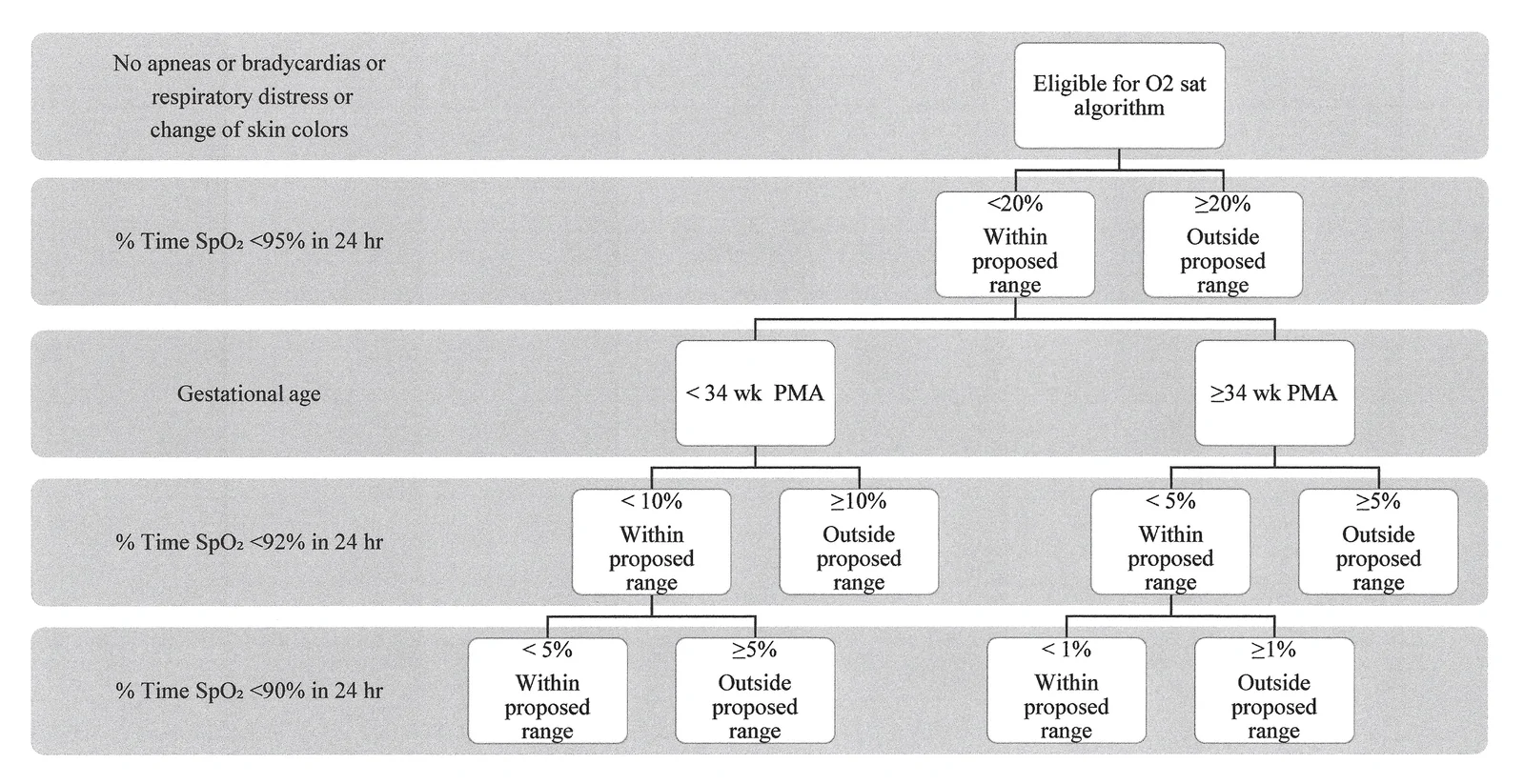
Proposed oxygen saturation threshold pathway for assessing oxygen weaning readiness, stratified by gestational age (hypothesis-generating; not validated). This is a proposed, hypothesis-generating decision pathway synthesized by the authors from the pattern identified across the included studies. It is presented separately from Table 4 and is not a visual restatement of Table 4's candidate parameters or their supporting citations, nor a validated clinical algorithm. At each step, infants whose values fall within the proposed range continue toward consideration of a room air trial; those outside the proposed range are flagged for continued monitoring or escalation of respiratory support, per clinical judgment. PMA, postmenstrual age; SpO₂, oxygen saturation.

Limitations

This review has several limitations. All eligible studies were observational; the retrospective design of studies such as Mohsen et al. (2022) may introduce bias from reliance on records not originally collected for research purposes [21], and variation in sample sizes limits statistical power and generalizability across NICU settings. Differences in inclusion and exclusion criteria across studies, for example, stark differences in gestational age and health status between the populations studied by Warburton et al. and Borenstein-Levin et al., may introduce variability in oxygen saturation responses that does not necessarily generalize to the broader NICU population [14,19]. Publication bias is also a concern, as studies with positive findings are more likely to be published than those with negative or inconclusive results. Reliance on data not originally collected for histogram-based research may have left unreported variables - differences in monitoring technology, oxygen-initiation criteria, or length of stay prior to room air trials - that could further limit generalizability. Although the modified QUIPS tool was used for quality assessment, some subjectivity is inherent to this process; independent assessment by two reviewers was used to mitigate this. Finally, substantial heterogeneity in study quality, design, patient populations, and prognostic factor definitions precluded meta-analysis, limiting this review to a narrative synthesis.

## Conclusions

This review asked whether trends in oxygen saturation histograms can predict readiness for room air trials or inform discharge criteria in NICU patients. Across nine included studies, more time spent within an approximate 90-95% saturation range was consistently associated with successful weaning, supporting histogram-based monitoring as a useful adjunct to clinical judgment. However, this evidence base is small, heterogeneous, and - critically for our stated aims - concentrated in preterm infants: only two studies directly addressed the late preterm and term population this review targeted. The candidate parameters proposed in the study should therefore be understood as hypothesis-generating starting points, not validated clinical guidelines. Because the studies did not evaluate sufficiently comparable exposure-outcome relationships using compatible definitions and effect measures, quantitative pooling was not methodologically appropriate. Future research should prioritize standardized histogram definitions and multicenter prospective data collection specifically in late preterm and term infants, with formal validation of any proposed thresholds before clinical adoption.

## Appendices

Supplementary Table

**Table 5:** Database-specific search strategies. Note: Searches were conducted between July and December 2024 and were limited to English-language studies published on or before November 30, 2024. Counts are the records returned or screened by each source before deduplication unless otherwise indicated. The Cochrane Library search returned 12 records; 10 were duplicates of records already identified through the other databases, and two were confirmed ineligible on inspection, so the Cochrane Library contributed no unique records for screening.

Database	Search date	Search strategy (Boolean)	Limits/Filters applied	Records retrieved
Embase	8-Jul-24	('histogram'/exp OR histogram:ab,ti,kw OR histograms:ab,ti,kw) AND ('oxygen'/de OR '16o':ab,ti,kw OR dioxygen:ab,ti,kw OR 'o 16':ab,ti,kw OR 'o 2':ab,ti,kw OR o2:ab,ti,kw OR oxigen:ab,ti,kw OR oxygen:ab,ti,kw OR 'oxygen saturation'/de OR 'o2 saturation':ab,ti,kw OR 'oxygen saturation':ab,ti,kw OR 'oxygen saturation index'/de OR 'weaning'/de OR wean:ab,ti,kw OR weaning:ab,ti,kw) AND ('infant'/exp OR 'prematurity'/exp OR newborn*:ab,ti,kw OR infant*:ab,ti,kw OR baby:ab,ti,kw OR babies:ab,ti,kw OR neonat*:ab,ti,kw OR 'pre matur*':ab,ti,kw OR prematur*:ab,ti,kw OR 'pre term':ab,ti,kw OR preterm:ab,ti,kw OR term:ab,ti,kw OR 'full term':ab,ti,kw OR premie:ab,ti,kw OR premies:ab,ti,kw)	English language; no other database limits applied.	60
MEDLINE/PubMed	15-Jul-24	((("neo-natal intensive care unit"[Title/Abstract] OR "neonatal ICU"[Title/Abstract] OR "newborn ICU"[Title/Abstract] OR NICU[Title/Abstract] OR NICUs[Title/Abstract]) OR (newborn*[Title/Abstract] OR infant*[Title/Abstract] OR baby[Title/Abstract] OR babies[Title/Abstract] OR neonat*[Title/Abstract] OR pre-term[Title/Abstract] OR preterm*[Title/Abstract] OR prematur*[Title/Abstract] OR term[Title/Abstract] OR "full term"[Title/Abstract] OR premie[Title/Abstract] OR premies[Title/Abstract] OR "Infant"[Mesh] OR "Infant, Premature"[Mesh])) AND (16o[Title/Abstract] OR dioxygen[Title/Abstract] OR "o 16"[Title/Abstract] OR "o 2"[Title/Abstract] OR o2[Title/Abstract] OR oxigen[Title/Abstract] OR oxygen[Title/Abstract] OR "o2 saturation"[Title/Abstract] OR "oxygen saturation"[Title/Abstract] OR SpO2[Title/Abstract] OR "peripheral oxygen"[Title/Abstract] OR oximetry[Title/Abstract] OR oximeter[Title/Abstract] OR oximeters[Title/Abstract] OR "Oxygen Saturation"[Mesh] OR "Oxygen"[Mesh:NoExp] OR "Oximetry"[Mesh:NoExp] OR "Ventilator Weaning"[Mesh] OR "respirator weaning"[Title/Abstract] OR "ventilation weaning"[Title/Abstract] OR "wean off mechanical respiration"[Title/Abstract] OR "wean off respiratory support"[Title/Abstract] OR "wean off vent support"[Title/Abstract] OR "weaning from mechanical ventilation"[Title/Abstract] OR "weaning of ventilation"[Title/Abstract] OR "weaning off mechanical ventilation"[Title/Abstract] OR "weaning off the ventilator"[Title/Abstract] OR "ventilator weaning"[Title/Abstract]) AND (histogram[Title/Abstract] OR histograms[Title/Abstract]))	English language; no other database limits applied.	58
CINAHL	15-Jul-24	(((TI histogram) OR (AB histogram) OR (TI histograms) OR (AB histograms)) AND (((MH "Oxygen Saturation") OR (MH "Oxygen") OR (MH "Pulse Oximetry") OR (MH "Oximetry") OR (MH "Oximeters+") OR (MH "Ventilator Weaning")) OR (TI (oxygen OR "oxygen saturation" OR SpO2 OR oximetry OR oximeter OR wean OR weaning)) OR (AB (oxygen OR "oxygen saturation" OR SpO2 OR oximetry OR oximeter OR wean OR weaning))) AND ((MH "Infant, Newborn+") OR (MH "Infant, Premature+") OR TI (newborn* OR infant* OR baby OR babies OR neonat* OR preterm* OR prematur* OR term OR "full term" OR premie OR premies OR NICU OR NICUs) OR AB (newborn* OR infant* OR baby OR babies OR neonat* OR preterm* OR prematur* OR term OR "full term" OR premie OR premies OR NICU OR NICUs)))	English language; no other database limits applied.	35
Web of Science	2-Aug-24	TS=((newborn* OR infant* OR baby OR babies OR neonat* OR "pre-term" OR preterm* OR prematur* OR term OR "full term" OR NICU OR NICUs) AND (oxygen OR "oxygen saturation" OR SpO2 OR "peripheral oxygen" OR oximetry OR oximeter OR wean OR weaning OR "ventilator weaning") AND (histogram OR histograms))	English language; no document-type or other database limits applied.	30
Cochrane Library	-	#1 MeSH descriptor: [Oxygen] explode all trees OR MeSH descriptor: [Oxygen Saturation] explode all trees OR MeSH descriptor: [Oximetry] this term only OR MeSH descriptor: [Ventilator Weaning] explode all trees OR (oxygen OR "oxygen saturation" OR SpO2 OR oximetry OR oximeter OR wean OR weaning):ti,ab,kw; #2 (histogram OR histograms):ti,ab,kw; #3 MeSH descriptor: [Intensive Care Units, Neonatal] this term only OR MeSH descriptor: [Infant] explode all trees OR MeSH descriptor: [Infant, Premature] explode all trees OR (newborn* OR infant* OR baby OR babies OR neonat* OR preterm* OR prematur* OR term OR "full term" OR NICU OR NICUs):ti,ab,kw; #4 #1 AND #2 AND #3	English language; no other database limits applied.	12 (0 unique)
Google Scholar	-	(histogram OR histograms OR "oxygen histogram") ("oxygen saturation" OR oxygen OR SpO2 OR oximetry OR "peripheral oxygen" OR wean OR weaning OR "ventilator weaning") (neonate OR neonatal OR newborn OR infant OR preterm OR prematurity OR premature OR term OR "full term" OR NICU)	English language; custom publication-date range ending November 30, 2024.	35 screened
Citation searching	-	Reference lists and citing articles of all studies retained after full-text review were screened manually for additional eligible English-language studies (backward and forward citation chasing).	Not applicable; citation searching was a supplementary screening method rather than a database search.	1 screened

## References

[1] Mascoll-Robertson KK, Viscardi RM, Woo HC (2016). The objective use of pulse oximetry to predict respiratory support transition in preterm infants: an observational pilot study. Respir Care.

[2] Gentle SJ, Ambalavanan N, Carlo WA (2020). Oxygen saturation histograms predict nasal continuous positive airway pressure-weaning success in preterm infants. Pediatr Res.

[3] Todd DA, Wright A, Broom M (2012). Methods of weaning preterm babies <30 weeks gestation off CPAP: a multicentre randomised controlled trial. Arch Dis Child Fetal Neonatal Ed.

[4] Gentle S, El-Ferzli G, Winter L, Salas AA, Philips Iii JB (2020). Oxygen saturation histogram monitoring to reduce death or retinopathy of prematurity: a quality improvement initiative. J Perinatol.

[5] Manja V, Saugstad OD, Lakshminrusimha S (2017). Oxygen saturation targets in preterm infants and outcomes at 18-24 months: a systematic review. Pediatrics.

[6] Montenegro B, Freiberger C, Veit L, Amberson M, Mukhopadhyay S, Rhein L (2018). An occurrence of apnea, bradycardia, and desaturation events resulting in a delay of discharge in late preterm and full term infants. Pediatr Neonatol.

[7] Poets CF, Roberts RS, Schmidt B (2015). Association between intermittent hypoxemia or bradycardia and late death or disability in extremely preterm infants. JAMA.

[8] Goetz EM, Tomlin BD, Rinaldo KE (2024). Oxygen saturation profiles in healthy term infants in early postnatal life. Am J Perinatol.

[9] Bachman TE, Roubik K (2022). Prevalence of potentially clinically relevant complex episodes of extreme SpO2 during manual and automatic control of inspired oxygen. Lek Tech.

[10] Afshar N, Baier RJ, Tagin M, Cabo C, Minski J, Elsayed YN (2018). The impact of hypoxemia on the development of retinopathy of prematurity in infants less than 29 weeks of gestation. Am J Perinatol.

[11] Borenstein-Levin L, Poppe JA, van Weteringen W (2023). Oxygen saturation histogram classification system to evaluate response to doxapram treatment in preterm infants. Pediatr Res.

[12] Ali SKM, Mohammed N, Qureshi N, Gupta S (2021). Oxygen therapy in preterm infants: recommendations for practice. Paediatr Child Health.

[13] Rafl J, Bachman TE, Martinek T, Tejkl L, Huttova V, Kudrna P, Roubik K (2019). Design and demonstration of a complex neonatal physiological model for testing of novel closed-loop inspired oxygen fraction controllers. World Congress on Medical Physics and Biomedical Engineering 2018. IFMBE Proceedings.

[14] Warburton A, Monga R, Sampath V, Kumar N (2019). Continuous pulse oximetry and respiratory rate trends predict short-term respiratory and growth outcomes in premature infants. Pediatr Res.

[15] Borenstein-Levin L, Haj A, Riskin A, Dinur G, Littner Y, Hochwald O, Kugelman A (2024). Oxygenation instability during bolus versus continuous feeding among very low birth weight premature infants, supported by noninvasive ventilation: a randomized prospective study. Am J Perinatol.

[16] Higgins JPT, Thomas J, Chandler J (2024). Cochrane Handbook for Systematic Reviews of Interventions. Version 6.5. https://www.cochrane.org/authors/handbooks-and-manuals/handbook/current.

[17] Page MJ, McKenzie JE, Bossuyt PM (2021). The PRISMA 2020 statement: an updated guideline for reporting systematic reviews. BMJ.

[18] Hayden JA, van der Windt DA, Cartwright JL, Côté P, Bombardier C (2013). Assessing bias in studies of prognostic factors. Ann Intern Med.

[19] Borenstein-Levin L, Konikoff L, Solimano A (2020). Clinical quantification of SpO2 instability using a new histogram classification system: a clinical study. Pediatr Res.

[20] Harigopal S, Satish HP, Taktak AF, Southern KW, Shaw NJ (2011). Oxygen saturation profile in healthy preterm infants. Arch Dis Child Fetal Neonatal Ed.

[21] Mohsen N, Nasef N, Elkhouli M (2022). Predictors of successful trial off continuous positive airway pressure and high flow nasal cannula in preterm infants <30 weeks' gestation: a retrospective study. Pediatr Pulmonol.

[22] Rath C, Kluckow M (2016). Oxygen saturation profile of term equivalent extreme preterm infants at discharge - comparison with healthy term counterparts. Acta Paediatr.

[23] Shah PS, Hakak H, Mohamed A, Shah J, Young J, Kelly E (2014). Oxygen saturation profile in late-preterm and term infants: a prospective cohort study. J Perinatol.

[24] Hunt CE, Corwin MJ, Lister G (1999). Longitudinal assessment of hemoglobin oxygen saturation in healthy infants during the first 6 months of age. J Pediatr.

[25] Samhouri B, Venkatasaburamini M, Paz Y Mar H, Li M, Mehra R, Chaisson NF (2020). Pulmonary artery hemodynamics are associated with duration of nocturnal desaturation but not apnea-hypopnea index. J Clin Sleep Med.

[26] Singh Y, Lakshminrusimha S (2021). Pathophysiology and management of persistent pulmonary hypertension of the newborn. Clin Perinatol.

[27] Duenas-Meza E, Escamilla-Gil MI, Bazurto-Zapata MA (2022). Intermittent hypoxia and respiratory patterns during sleep of preterm infants aged 3 to 18 months residing at high altitude. Sleep.

[28] Di Fiore JM, Wilson-Costello D, Chen Z, Minich NM, Martin RJ, Hibbs AM (2025). Intermittent hypoxemia and neurodevelopmental impairment at 12 and 24 months in preterm infants. Neonatology.

[29] Sur A, Paria A (2021). Histogram analysis for bedside respiratory monitoring in not critically ill preterm neonates: a proposal for a new way to look at the monitoring data. Eur J Pediatr.

[30] Guo F, Tang S, Guo T, Bartell S, Detrano R (2020). Revised threshold values for neonatal oxygen saturation at mild and moderate altitudes. Acta Paediatr.

[31] Luks AM, Hackett PH (2022). Medical conditions and high-altitude travel. N Engl J Med.

